# Effects of cold environment exposure on female reproductive health and its regulatory mechanisms

**DOI:** 10.3389/fgene.2025.1570053

**Published:** 2025-04-09

**Authors:** Hongyi Sun, Qianqian Zhao, Xiaolan Liang, Yalun He, Yangshuo Li, Jin Yu, Jie Ding, Chaoqin Yu

**Affiliations:** ^1^ Basic Medicine School, Naval Medical University, Shanghai, China; ^2^ Department of Traditional Chinese Gynecology, The First Affiliated Hospital of Naval Medical University, Shanghai, China; ^3^ Department of Physiotherapy of Traditional Chinese Medicine, Beidaihe Rehabilitation and Recuperation Center, The People’s Liberation Army Joint Logistic Support Force, Qinhuangdao, China

**Keywords:** cold, female, reproduction, sex hormones, inflammation

## Abstract

**Objective:**

To investigate the effects of cold environment exposure on female reproductive capacity and explore its potential regulatory mechanisms.

**Methods:**

Female mice were subjected to cold water immersion to simulate cold environment exposure. Weight changes during cold exposure were recorded. Serum levels of anti-Müllerian hormone (AMH), estradiol (E2), follicle-stimulating hormone (FSH), and luteinizing hormone (LH) were measured using enzyme-linked immunosorbent assay (ELISA). Ovarian and uterine tissues were collected via surgical procedures, and transcriptomic sequencing was performed to explore potential regulatory mechanisms. ELISA was used to assess the levels of inflammatory cytokines, including interleukin-1β (IL-1β), interleukin-6 (IL-6), interleukin-18 (IL-18), and tumor necrosis factor-alpha (TNF-α) in peritoneal fluid. Furthermore, immunohistochemistry was used to detect the expression levels of IL-1, IL-6, and IL-18 in ovarian tissues, as well as IL-6 and IL-18 in uterine tissues.

**Results:**

Compared with the control group, female mice exposed to cold environments exhibited a significant increase in body weight and elevated serum levels of AMH, E2, FSH, and LH. Transcriptomic sequencing of ovarian and uterine tissues indicated that differentially expressed genes were primarily enriched in inflammation-related pathways, including the cAMP signaling pathway, cytokine-cytokine receptor interaction, and PI3K-Akt signaling pathway. Additionally, levels of inflammatory cytokines in the peritoneal fluid, including IL-1β, IL-6, IL-18, and TNF-α, were significantly elevated. Immunohistochemical analysis showed that the expression levels of IL-1, IL-6, and IL-18 were markedly increased in ovarian tissue, while IL-6 and IL-18 expression levels were significantly elevated in uterine tissue. These differences were statistically significant (P < 0.05).

**Conclusion:**

Cold environment exposure may induce inflammatory responses in the uterus and ovaries, contributing to the formation of an inflammatory microenvironment in the reproductive system. This process may lead to disruptions in sex hormone levels and ultimately impair female reproductive capacity.

## 1 Introduction

An optimal environmental temperature is essential for maintaining human health. Extensive epidemiological studies have highlighted that fluctuations in environmental temperature can increase morbidity and mortality risks, exerting profound negative effects on human health (Cheng et al., 2019; Cheng et al., 2018; Fu et al., 2018). Among these health risks, female reproductive disorders represent a characteristic consequence of cold exposure (Chan et al., 2001; Wang et al., 2020). However, the mechanisms underlying cold-induced female reproductive dysfunction remain unclear.

Previous studies have reported that cold environment exposure significantly affects the female reproductive system in rodents. Specifically, cold stimulation disrupts the estrous cycle and impairs ovarian structure and function. Research has shown (Xu et al., 2018) that cold exposure leads to a reduction in the diameter of the granulosa and theca cell layers within the ovary, accompanied by a decline in follicle numbers. Serum levels of follicle-stimulating hormone (FSH) and luteinizing hormone (LH) were markedly elevated. Additionally, cold exposure has been found to increase serum progesterone (P) levels while upregulating progesterone receptor (PR) expression and downregulating estrogen receptor (ER) expression in the uterus. These hormonal changes result in uterine structural damage, including a reduction in the uterine index, glandular expansion, and significant thinning of the endometrial layer, ultimately impairing normal uterine function (Zhao et al., 2019).

Moreover, environmental temperature is closely associated with systemic inflammation. Studies on the digestive, respiratory, and cardiovascular systems have consistently demonstrated that cold exposure elevates inflammatory levels (Guo et al., 2022; Liu et al., 2023; Wei et al., 2024). However, research on the impact of cold exposure on reproductive inflammation remains limited. In this study, transcriptomic sequencing of the ovarian and uterine tissues following cold exposure was performed to investigate the effects of cold exposure on uterine and ovarian inflammation. By exploring the potential mechanisms underlying cold-induced reproductive dysfunction, we propose that cold exposure alters the inflammatory microenvironment of the reproductive system, leading to female reproductive disorders.

## 2 Materials and methods

### 2.1 Animal model establishment

Ten healthy 8-week-old female SPF-grade C57 nude mice (Zhejiang Weitonglihua Experimental Animal Technology Co., Ltd., SCXK (Zhe) 2019-0001), weighing 22 ± 1 g, were housed at the Chang Hai Hospital Experimental Animal Center with free access to food and water. After 1 week of acclimatization, the mice were randomly divided into two groups (n = 5 per group): the control group and the cold exposure model group.

Cold exposure was simulated by immersing the mice in cold water (4°C) daily at 15:00, with the water level reaching the neck while ensuring unobstructed breathing. Each immersion lasted for 6 minutes. After exposure, the mice were immediately removed from the water, dried with a towel, and dried with a hair dryer to prevent hypothermia-induced mortality. The procedure was repeated for 21 consecutive days, and body weight was recorded every 3 days. At the end of the experiment, blood samples were collected via eyeball enucleation, and the mice were euthanized by cervical dislocation. The ovaries, uterus, and peritoneal lavage fluid were collected for subsequent analysis.

### 2.2 Serum sex hormone analysis

The collected blood samples were centrifuged at 3000 rpm for 15 min at 4°C to separate the serum. Serum levels of anti-Müllerian hormone (AMH), estradiol (E2), follicle-stimulating hormone (FSH), and luteinizing hormone (LH) were measured using enzyme-linked immunosorbent assay (ELISA) kits (Elabscience, Wuhan, China), following the manufacturer’s instructions.

### 2.3 Transcriptomic analysis

Transcriptomic sequencing of ovarian and uterine tissues was performed using the fragments per kilobase of transcript per million mapped reads (FPKM) method. Genes with significant differential expression were identified based on the criteria of *p* < 0.05 and fold change >2. Volcano plots and heat maps were generated to visualize the distribution of differentially expressed genes, and hierarchical clustering was used to illustrate the expression patterns. Gene Ontology (GO) analysis was conducted to examine gene enrichment in biological processes (BP), cellular components (CC), and molecular functions (MF). Kyoto Encyclopedia of Genes and Genomes (KEGG) pathway analysis was performed to identify regulatory signaling pathways.

### 2.4 Inflammatory cytokine analysis in peritoneal fluid

The peritoneal lavage fluid was centrifuged at 1,500 rpm for 15 min at 4°C, and the supernatant was collected. The levels of inflammatory cytokines, including interleukin-1β (IL-1β), interleukin-6 (IL-6), interleukin-18 (IL-18), and tumor necrosis factor-alpha (TNF-α), were measured using ELISA kits (Elabscience, Wuhan, China) following the manufacturer’s protocol.

### 2.5 Immunohistochemical staining analysis

Paraffin-embedded ovarian and uterine tissue sections were deparaffinized, and endogenous peroxidase activity was quenched. Antigen retrieval was performed using citric acid buffer. The sections were blocked with 5% bovine serum albumin (BSA) at 37°C for 30 min, followed by incubation with primary antibodies overnight at 4°C. The sections were then incubated with secondary antibodies at 37°C for 30 min and visualized using 3,3′-diaminobenzidine (DAB). The samples were dehydrated, sealed with neutral resin, and examined under a microscope.

### 2.6 Statistical analysis

All data are presented as means ± standard error (SE). Statistical analysis was conducted using the SPSS software (version 26.0). An independent *t*-test was used to compare differences between groups, with *p* < 0.05 considered statistically significant. Prior to conducting the *t*-test, all datasets underwent normality testing and homogeneity of variance testing.

## 3 Results

### 3.1 Effects of cold exposure on mouse body weight

**FIGURE 1:**
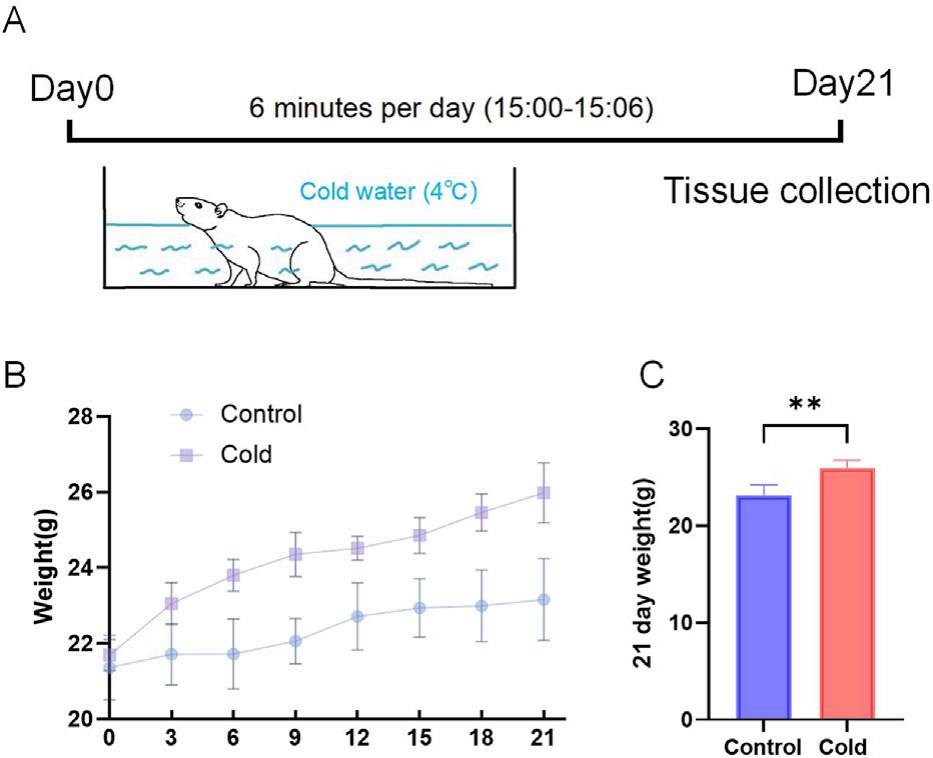
Animal Model Establishment and body weight. **(A)** Schematic representation of the cold exposure model in mice. **(B)** Body weight variation curves for each group. **(C)** Final body weights of the mice at the end of the experiment. **P < 0.01.

Cold exposure was simulated by subjecting the experimental group of mice to cold water immersion (Figure 1A). Body weight was measured every 3 days, and a body weight variation curve was plotted (Figure 1B). At the end of the experiment, the final body weight of the mice in each group was recorded (Figure 1C).

### 3.2 Effects of cold exposure on serum sex hormone levels in female mice

**FIGURE 2:**
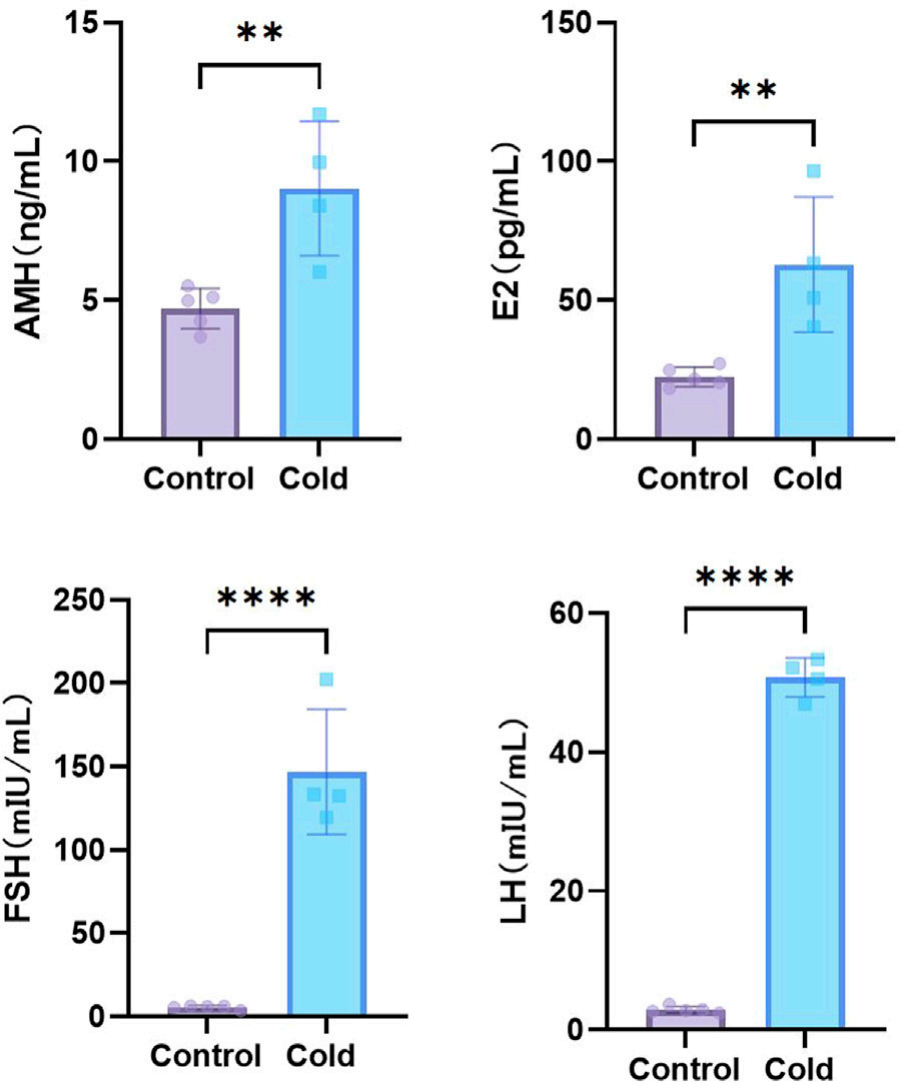
Levels of sex hormones of mice in each group. **P < 0.01, ****P < 0.0001.

The results showed that after simulated cold exposure, serum levels of anti-Müllerian hormone (AMH), estradiol (E2), follicle-stimulating hormone (FSH), and luteinizing hormone (LH) were significantly elevated in female mice compared with those in the control group (Figure 2).

### 3.3 Transcriptomic sequencing results

**FIGURE 3:**
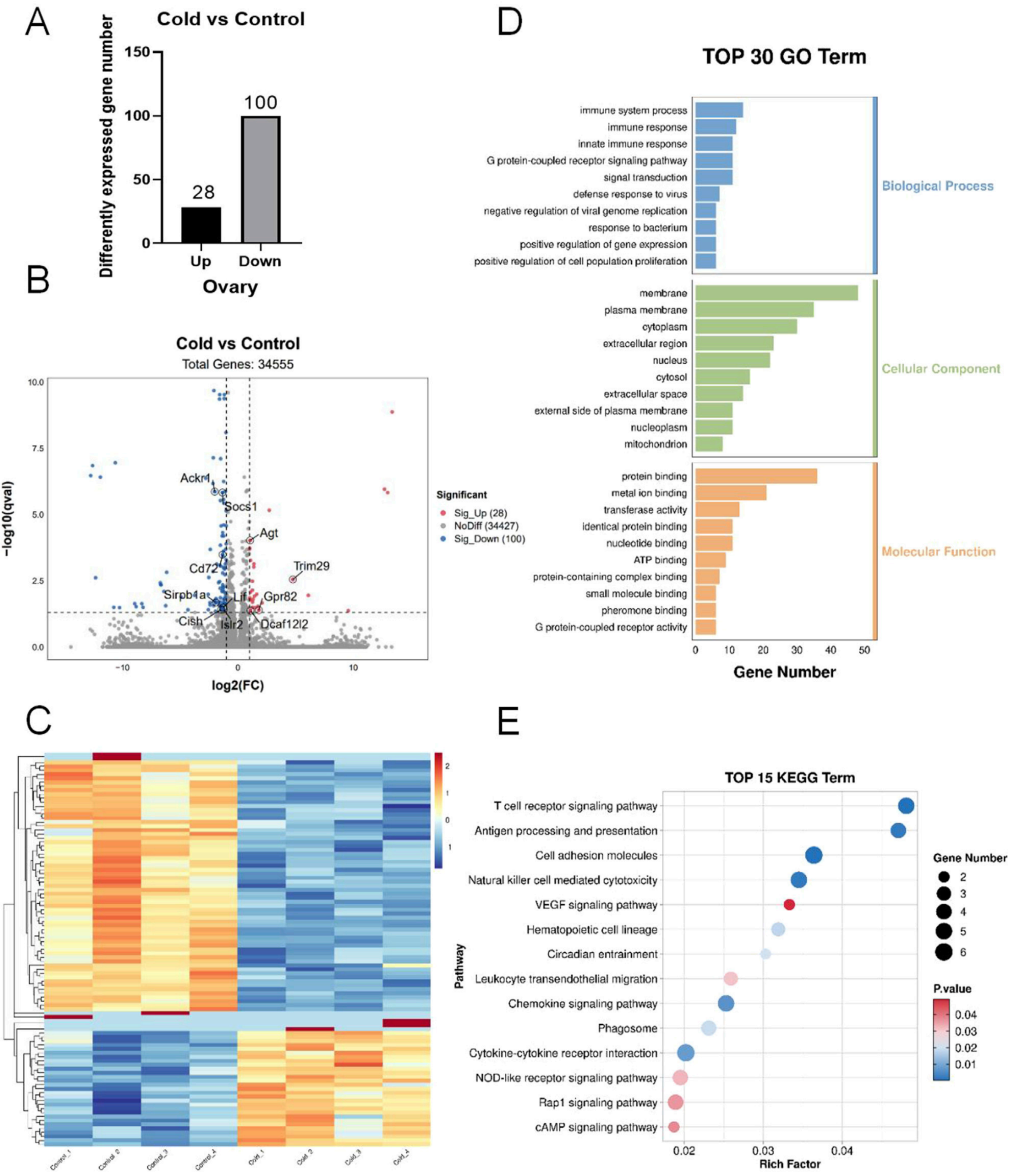
Transcriptomic sequencing of mouse ovarian tissues. **(A)** Number of differentially expressed genes (DEGs). **(B)** Volcano plot of the DEGs. **(C)** Heatmap of the DEGs. **(D)** Gene Ontology (GO) enrichment analysis of DEGs. **(E)** Kyoto Encyclopedia of Genes and Genomes (KEGG) pathway enrichment analysis of the DEGs.

Transcriptomic sequencing of ovarian tissues from cold-exposed mice revealed 28 upregulated genes and 100 downregulated genes (Figure 3A). The distribution of differentially expressed genes (DEGs) was visualized using a volcano plot (Figure 3B) and heatmap (Figure 3C). Several DEGs, including *Agt, Trim29, Socs1, Cish, Cd72, Ackr1, Lif, Islr2,* and *Sirpb1a*, were closely associated with inflammation. Gene Ontology (GO) enrichment analysis indicated that biological processes (BP) were mainly related to immune responses, G-protein-coupled receptor signaling pathways, and signal transduction. The cellular component (CC) category showed significant enrichment in the membrane, plasma membrane, cytoplasm, and nucleus. The molecular function (MF) category was predominantly enriched in protein binding, metal ion activity, transferase activity, homodimerization activity, and ATP binding (Figure 3D). Kyoto Encyclopedia of Genes and Genomes (KEGG) pathway analysis showed significant enrichment in inflammation-related pathways, including the VEGF signaling pathway, leukocyte transendothelial migration, cAMP signaling pathway, cytokine-cytokine receptor interaction, Rap1 signaling pathway, and NOD-like receptor signaling pathway (Figure 3E).

**FIGURE 4:**
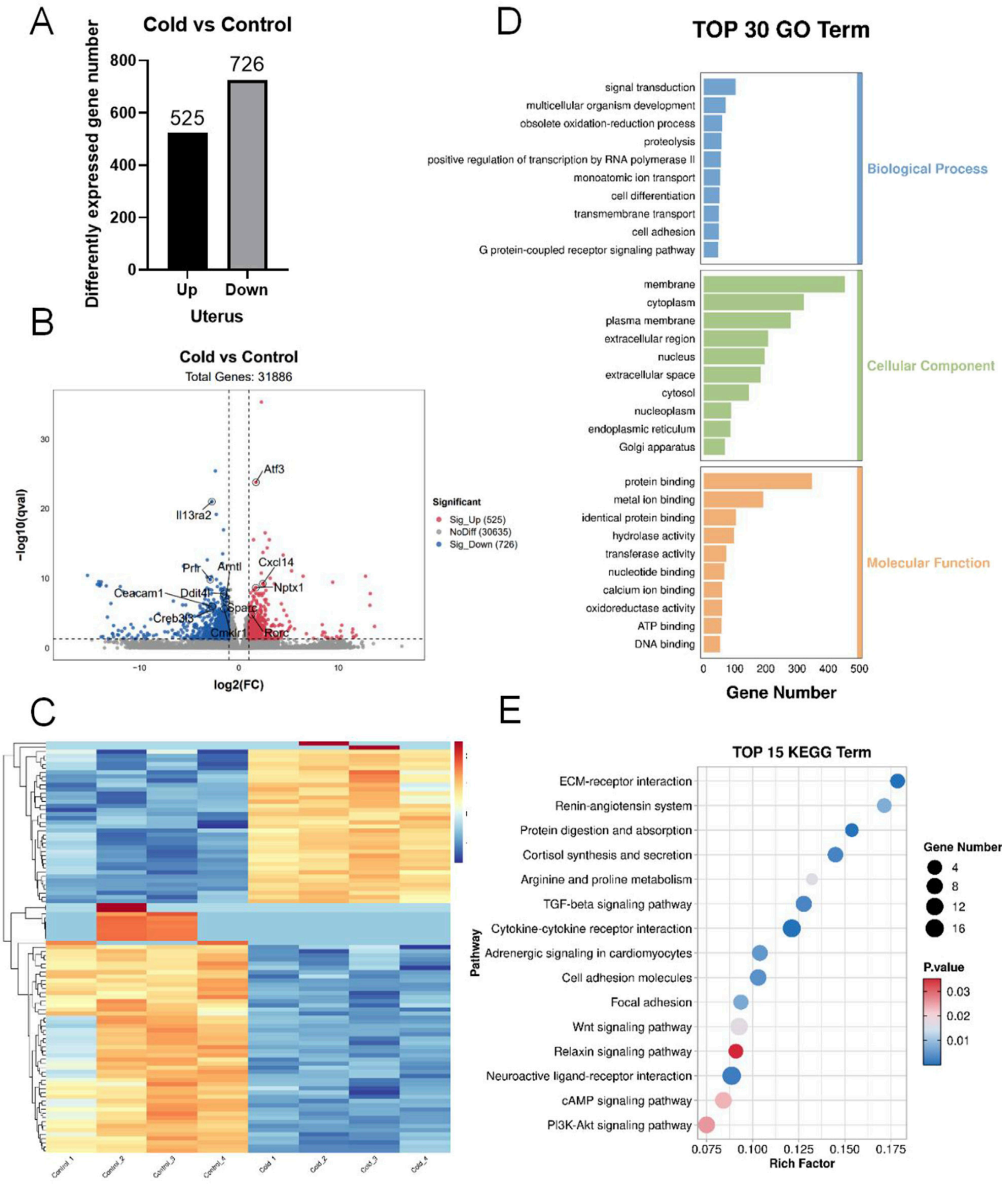
Transcriptomic sequencing of mouse uterine tissues. **(A)** Number of differentially expressed genes (DEGs). **(B)** Volcano plot of the DEGs. **(C)** Heatmap of the DEGs. **(D)** Gene Ontology (GO) enrichment analysis of DEGs. **(E)** Kyoto encyclopedia of genes and genomes (KEGG) pathway enrichment analysis of the DEGs.

Transcriptomic sequencing of uterine tissues after cold exposure identified 525 upregulated genes and 726 downregulated genes (Figure 4A). The distribution of DEGs is shown in the volcano plot (Figure 4B) and the heatmap (Figure 4C). The key DEGs related to inflammation included *Nptx1, Atf3, Cxcl14, Rorc, Arntl, Cmklr1, Ceacam1, Creb3l3, Sparc, Prlr, Il13ra2*, and *Ddit4l*. GO enrichment analysis indicated that biological processes (BP) were primarily associated with signal transduction, redox processes, proteolysis, and immune responses. The cellular component (CC) category was enriched in the membrane, plasma membrane, cytoplasm, and nucleus. The molecular function (MF) category showed predominant enrichment in protein binding, metal ion activity, transferase activity, homodimerization activity, and ATP binding (Figure 4D). KEGG pathway analysis revealed significant enrichment in inflammation-related pathways, including the relaxin signaling pathway, PI3K-Akt signaling pathway, cAMP signaling pathway, cytokine-cytokine receptor interaction, Wnt signaling pathway, and TGF-β signaling pathway (Figure 4E).

### 3.4 Effects of cold exposure on inflammatory levels in female mice

**FIGURE 5:**
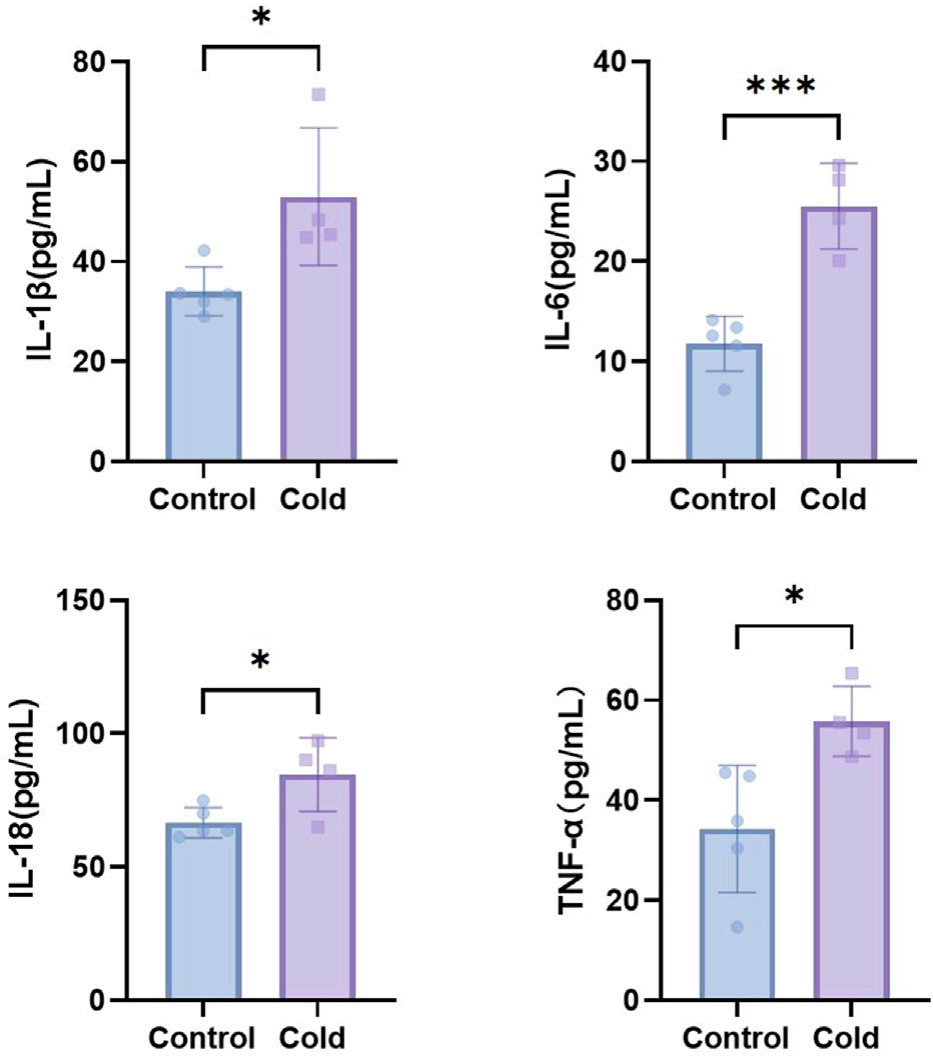
The levels of IL-1β, IL-6, IL-18, and TNF-α in the intraperitoneal irrigation fluid of mice in each group. *P < 0.05, ***P < 0.001.

**FIGURE 6:**
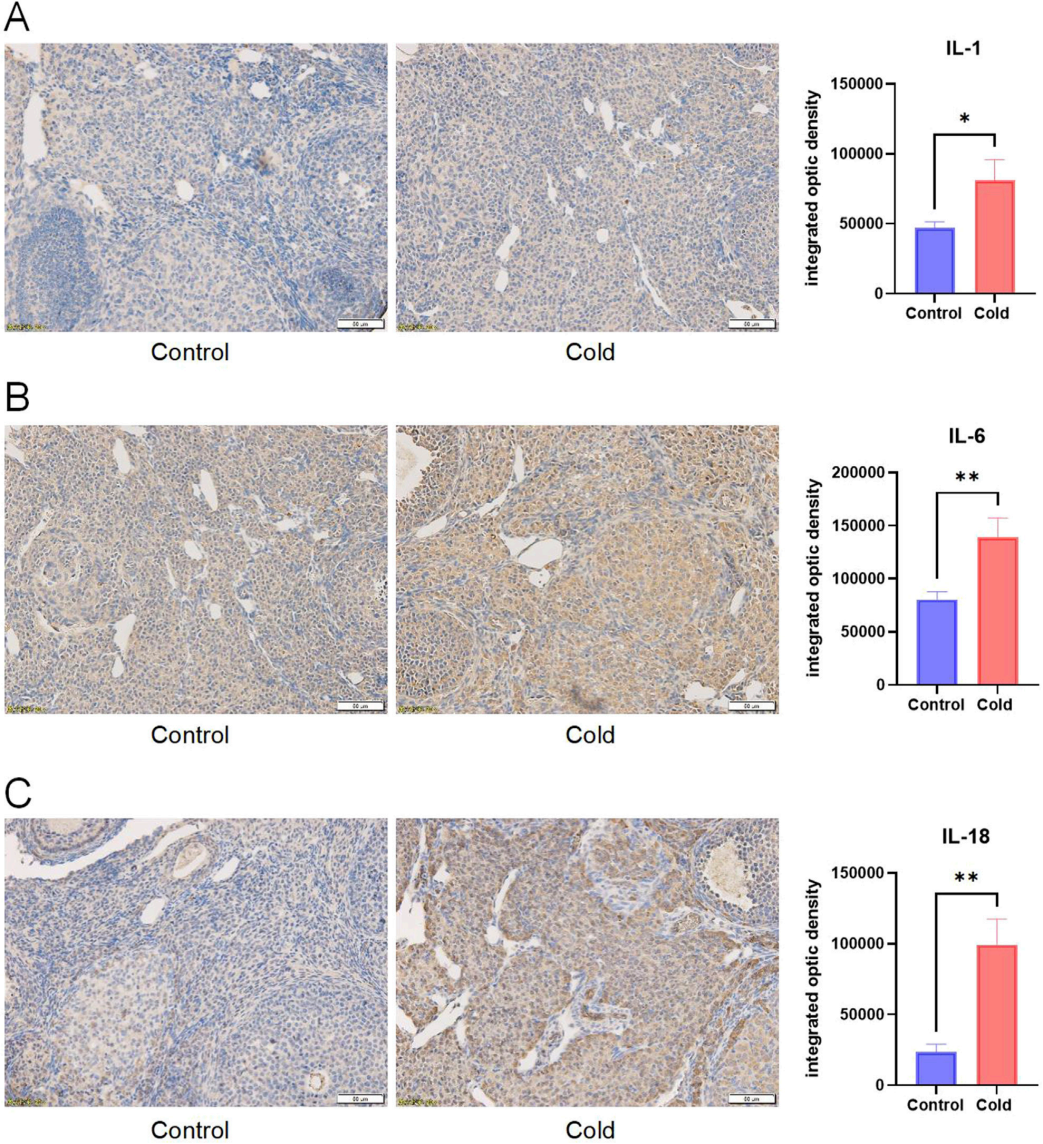
Representative immunostaining and levels of inflammatory cytokines IL-1 **(A)**, IL-6 **(B)**, and IL-18 **(C)** in the ovarian tissues of mice in each group. Scale bar = 50μm. * P < 0.05, ** P < 0.01.

**FIGURE 7:**
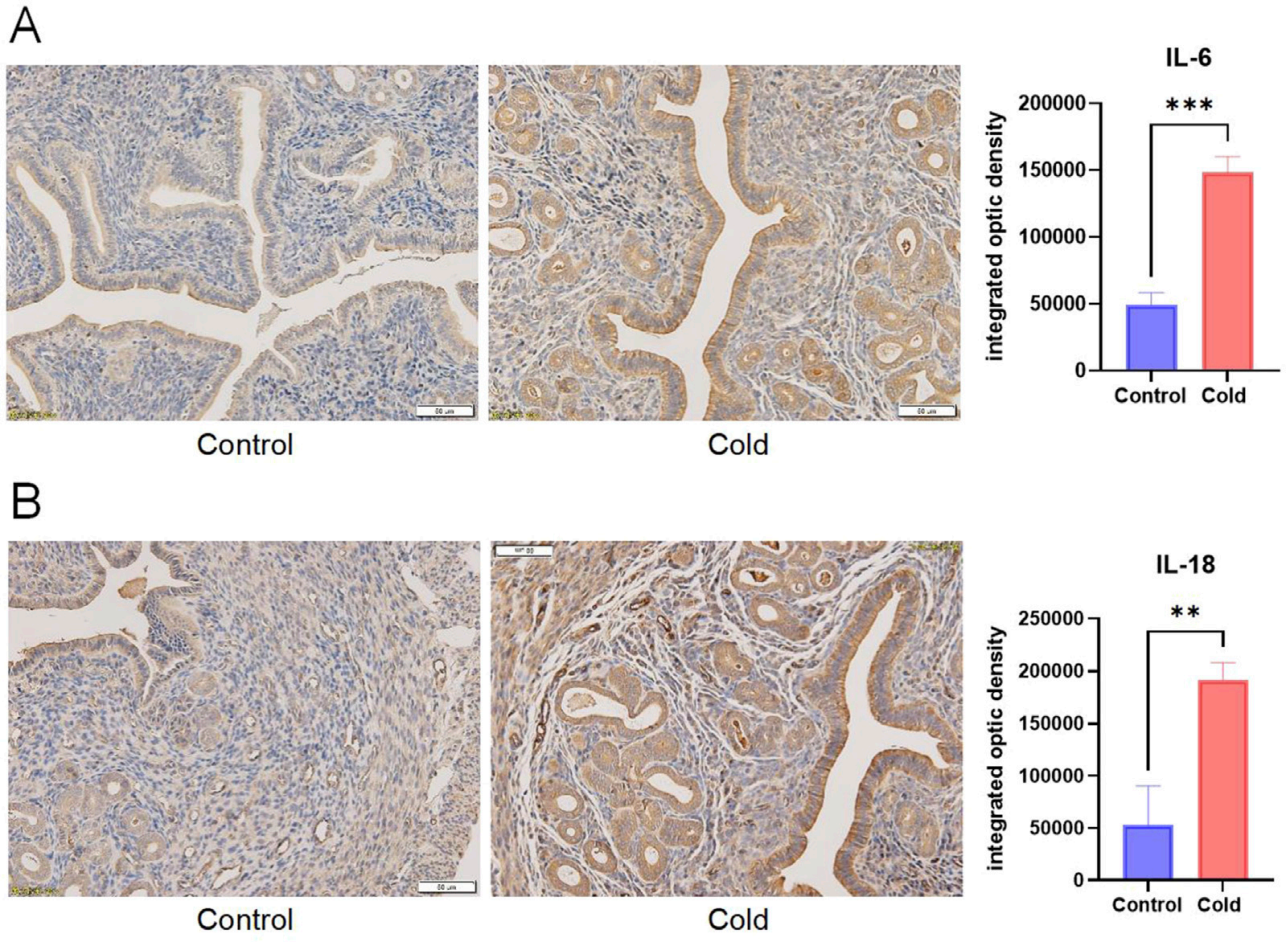
Representative immunostaining and levels of inflammatory cytokines IL-6 **(A)**, and IL-18 **(B)** in the uterine tissues of mice in each group. Scale bar = 50μm. ** P < 0.01, *** P < 0.001.

Compared with the control group, cold-exposed mice exhibited significantly elevated levels of inflammatory cytokines IL-1β, IL-6, IL-18, and TNF-α in the intraperitoneal irrigation fluid (Figure 5). Immunohistochemical analysis revealed increased expression levels of IL-1, IL-6, and IL-18 in the ovarian tissues of cold-exposed mice compared those to in the control group (Figure 6). Similarly, IL-6 and IL-18 expression levels were elevated in the uterine tissues (Figure 7).

## 4 Discussion

Cold exposure is a critical environmental factor influencing reproductive function, representing an adaptive response of animals and humans to external and internal environmental stimuli (Varela et al., 2015; Brazaitis et al., 2015). This study demonstrated that cold exposure elevated serum sex hormone levels and induced inflammation in the uterus and ovaries of female mice, suggesting that cold exposure may promote the formation of an inflammatory microenvironment in these reproductive organs, ultimately impairing reproductive function. These findings provide preliminary insights into the regulatory mechanisms by which cold environments affect female reproductive function.

Our results revealed a significant increase in body weight gain in cold-exposed mice. Previous studies have shown that cold exposure affects mammalian energy metabolism (Zhang et al., 2021) by increasing the basal metabolic rate due to heightened energy expenditure (Westerterp-Plantenga et al., 2002). In this study, food intake was not restricted, and research suggests that cold exposure tends to enhance appetite in mammals (Langeveld et al., 2016), leading mice to consume more food to meet their increased energy demands and maintain body temperature (Author anonymous, 2023) (McInnis et al., 2020). Excessive food intake may exceed energy expenditure, leading to fat accumulation. Aldiss et al. (2022) found that cold exposure promotes subcutaneous fat deposition in mice, an adaptive mechanism that enhances thermal insulation and contributes to weight gain, which aligns with our observations. Increased fat accumulation and body weight may significantly elevate the risk of obesity in females, which is known to have adverse effects on reproductive function, including ovulation and menstrual cycle irregularities, reduced fertility, lower success rates and safety concerns in infertility treatments, and potential negative impacts on offspring health (Practice Committee of the American Society for Reproductive MedicinePractice Committee of the American Society for Reproductive Medicine, 2021; Armstrong et al., 2022; Garg et al., 2023).

We further investigated the effects of cold exposure on female reproductive function. Compared to the control group, cold-exposed mice exhibited elevated serum levels of AMH, E2, FSH, and LH. Ovarian and uterine reproductive functions are tightly regulated by sex hormones. E2 facilitates follicular development and uterine contractions, FSH promotes follicular maturation, and prepares the endometrium for implantation, while LH supports corpus luteum development and stimulates ovulation. AMH is a key indicator of ovarian reserve and plays a role in regulating gonadotropin and estrogen levels (di Clemente et al., 2021). Previous studies have shown that cold exposure disrupts sex hormone balance and induces female reproductive dysfunction (Macalpine et al., 2011). Cold exposure activates the PERK/NRF2 pathway, upregulating CX4 expression, and leading to StAR-dependent progesterone elevation in ovarian granulosa cells. This results in increased progesterone levels, estrous cycle disruption, and impaired follicular development, ultimately contributing to reproductive dysfunction (Ding et al., 2024). Xu et al. (2018) reported that female rats exposed to −10°C for 2 weeks exhibited increased serum LH levels, suggesting that cold exposure may impair ovarian function by altering LH levels and upregulating LHR expression. Another study (Zhang et al., 2021) found that rats exposed to cold conditions for 2 weeks exhibited increased FSH and E2 levels but decreased AMH levels, potentially due to dysregulation of the hypothalamic-pituitary-ovarian (HPO) axis, which could lead to ovarian dysfunction and impaired follicular development. The elevated AMH levels observed in our study may reflect ovarian stress response to cold exposure. Additionally, increased FSH levels can stimulate E2 production (Liu et al., 2021), potentially explaining the simultaneous increase in FSH and E2 levels. However, the interactions among these hormones, along with the regulatory control from the hypothalamus and pituitary gland, remain unclear. We only observed sex hormone dysregulation in female mice, the underlying regulatory mechanisms require further investigation.

Previous studies have reported that cold exposure increases systemic inflammatory cytokines such as IL-1β, IL-6, and TNF-α in patients with type 2 diabetes (Cai et al., 2016). Teng et al. (2024) observed Toll-like receptor 4 (TLR4) pathway activation and enrichment of IL-1β and TNF-α in the liver and pancreas of cold-exposed pigs, indicating a strong correlation between cold exposure and systemic inflammation. Moreover, obesity is often accompanied by a state of chronic low-grade inflammation (Denizli et al., 2022). Transcriptomic sequencing of ovarian and uterine tissues in cold-exposed mice revealed significant enrichment of DEGs in inflammation-related pathways, including the cAMP signaling pathway, cytokine-cytokine receptor interaction, and PI3K-Akt signaling pathway (Xie et al., 2023; Acosta-Martinez and Cabail, 2022). The cAMP signaling pathway is implicated in the inflammatory activation of human myometrial cells, potentially triggering preterm labor (Riaposova et al., 2023). Pro-inflammatory cytokines and their receptors can impair endometrial receptivity (Ishikawa et al., 2024), and activation of the PI3K-Akt signaling pathway has been identified as a key contributor to endometrial inflammatory changes in patients with endometriosis (An et al., 2024). These findings suggest that inflammation plays a crucial role in cold-induced female reproductive dysfunction.

Further analysis of intraperitoneal irrigation fluid revealed significantly elevated levels of IL-1β, IL-6, IL-18, and TNF-α in cold-exposed mice, indicating the formation of an inflammatory microenvironment. Immunohistochemical analysis of ovarian and uterine tissues demonstrated increased expression of IL-6 and IL-18 in cold-exposed mice compared to the control group. IL-1β, a member of the IL-1 cytokine family, is primarily produced by monocytes and macrophages and plays a crucial role in the host defense against infection and injury (Lopez-Castejon and Brough, 2011). IL-18, another IL-1 family member, is known for its ability to induce IFN-γ production and plays a regulatory role in mucosal and systemic inflammation (Landy et al., 2024). IL-6, secreted by T cells, is a key mediator of immune and inflammatory responses (Choy et al., 2020), and exhibits both pro-inflammatory and anti-inflammatory properties depending on environmental conditions (Hunter and Jones, 2015). Tumor necrosis factor-alpha (TNF-α) is a multifunctional Th1 cytokine and major inflammatory mediator. Elevated levels of IL-1β, IL-6, IL-18, and TNF-α in our study indicate that cold exposure induces inflammatory changes in the reproductive system, which is consistent with previous findings that chronic cold stress promotes the release of TNF-α, IL-1β, IL-18, and IL-6 in the ileum, leading to intestinal injury (Lv et al., 2024). Therefore, we conclude that cold exposure induces inflammation of the reproductive system, leading to ovarian and uterine damage, which may further impair female reproductive function. Additionally, reproductive inflammation can contribute to the pathogenesis of disorders such as polycystic ovary syndrome (PCOS) and endometriosis, exacerbating female reproductive dysfunction (Guo et al., 2024).

However, this study had several limitations. First, our investigation was limited to phenotypic analysis, with transcriptomic sequencing primarily focusing on the inflammatory responses. We only assessed tissue and fluid cytokine levels, without conducting mechanistic studies. Second, our conclusions were based on cold water immersion, which simulates a low-temperature, high-humidity environment. The applicability of our findings to different conditions, such as high-altitude cold environments, cold and dry desert climates, and aerospace conditions, remains uncertain and requires further investigation in diverse settings to enhance reliability. Finally, this study lacks clinical validation, and future research should collect data from reproductive-aged women working in cold environments to provide more comprehensive insights.

In conclusion, our study demonstrated that cold exposure induces inflammatory changes in the uterus and ovaries, promoting the formation of an inflammatory microenvironment in the reproductive system and impairing female reproductive function. These findings offer new perspectives for the prevention and treatment of reproductive disorders in extreme environmental conditions.

## Data Availability

The datasets presented in this study can be found in online repositories. The names of the repository/repositories and accession number(s) can be found in the article/supplementary material.
